# Acute Rheumatism

**Published:** 1875-04

**Authors:** 


### ACUTE RHEUMATISM.

Professor N. S. Davis (*Chicago Med. Examiner*) gives the following treatment:

Whether it is lactid acid, as claimed by Richardson, and suggested by many others, can hardly be considered fully determined. Conceding this theory of the pathology of the disease to be correct, the indications for treatment become obvious. They are first, to neutralize the excess of acid in the system by the free use of the non-purgative alkaline salts; second, to mitigate the patient's suffering; and third, to lessen the plasticity of the exudative material, in the inflamed parts, and hasten its absorption. Perhaps the best means for accomplishing the first of these objects, is to give the patient 20 grains of the carbonate, or 30 grains of bicarbonate of potassa, dissolved in plenty of water, every two or three hours, and an occasional dose of the Rochelle salts, to move the bowels. In moderate cases, the second indication is sufficiently met by a full dose of pulv. Dover's, with a grain or two of calomel, at bed-time each night. But in the more acute and severe cases, a teaspoonful of the following prescription, given between each of the doses of alkaline salts, during the first two or three days, will aid very much, both in mitigating the pain and lessening the fever: ℞. Nitrous ether, ℥j.; vin colchicum, ℥j·; camph. tinct. opii, ℥jj.; tinct. verat. viride, ℥j. M.

After the skin has become moist, the urine more free and less acid, the pulse softer and less frequent, and the inflammation has ceased to extend to new articulations, this sedative mixture may be omitted, and the patient left on the use of the alkaline salts alone, with only a dose of Dover's powder, without the calomel, at night. If, at the end of one week or more, the fever has subsided, leaving the patient pale, the skin relaxed and almost constantly bathed with perspiration, and yet the joints stiff and weak, from two to three grain doses of quinine, given three times a day, will generally be of much service.

Local applications to the inflamed articulations are generally of but little importance; yet, in the acute stages, keeping the part constantly wrapped in cloths wet in an infusion of aconite leaves, holding in solution hydrochlorate of ammonia, will aid much to mitigate the pain.

Such is the general outline of treatment that has been found most efficacious in the treatment of acute rheumatism. When resorted to early and followed pursued, it will, in many cases, lead to convalescence in from seven to ten days; and there will occur a less ratio of cardiac complications than under any other system of treatment that has yet been tried.

There are some cases, however, that persist through a period of three, four, or even six weeks, despite of all treatment. There are many other articles of the materia medica that may be used instead of those already named for fulfilling the same indications. Thus, the bitartrate of potassa may be used instead of the carbonates; or the salts of soda may be substituted for those of potassa; or the oxide of calcium, known in the shops as the syrup of lime, may be used. And in cases where the colchicum purges the bowels too much, it may be replaced with double of its quantity of tincture of cimcifuga rac.

